# High seroprevalence of hepatitis E virus in the ethnic minority populations in Yunnan, China

**DOI:** 10.1371/journal.pone.0197577

**Published:** 2018-05-22

**Authors:** Yue Feng, Yue-Mei Feng, Songmei Wang, Fang Xu, Xuehui Zhang, Chunyue Zhang, Yuanyuan Jia, Wanru Yang, Xueshan Xia, Jianzhong Yin

**Affiliations:** 1 Faculty of Life Science and Technology, Kunming University of Science and Technology, Kunming, Yunnan, China; 2 Research Institutes of Nutrition and Food Science, Kunming Medical University, Kunming, Yunnan, China; 3 Yunnan Key Laboratory of Nutrition and Food Safety, Kunming, Yunnan, China; Centers for Disease Control and Prevention, UNITED STATES

## Abstract

Hepatitis E virus (HEV) infection is relatively high in the southern regions of China. Yunnan, located in southwestern China, has the highest number of ethnic groups. However, HEV infection in the ethnic population is largely unknown. Therefore, we aimed to investigate the seropositive rate, risk factor, and clinical impact of HEV infection in the ethnic groups of Yunnan. We recruited 1912 individuals from four minority groups in three prefectures of Yunnan province. Epidemiological records on potential risk factors for exposure to HEV and blood biochemical index were analyzed. All the serum samples were tested for anti-HEV IgM/IgG by enzyme-linked immunosorbent assay, and the IgM-positive samples were subjected to nested reverse transcription-PCR to detect HEV RNA. Overall, 1273 individuals (66.58%) were positive for anti-HEV IgG, 16 (0.84%) for anti-HEV IgM, and 64 (3.35%) for anti-HEV IgG and IgM both; none of them had detectable HEV RNA. Multivariate analysis revealed a strong statistical association between ethnic origin and HEV IgG seroprevalence. Anti-HEV IgG reactivity in the Hani ethnic (82.3%; 401/487) population was higher than that in the Naxi (71.9%, 340/473), Bulang (65.1%; 302/464), and Wa (60.2%; 294/488) ethnic populations (p < 0.0001). Older age and male sex were independently associated with the risk of past HEV infection. Moreover, anti-HEV IgG-positive individuals showed significantly higher levels of total and direct bilirubin and alanine amino transferase but significantly lower levels of globulin and low-density lipoprotein, than the respective levels in anti-HEV IgG-negative individuals. Thus, the seroprevalence of HEV infection is high in the ethnic populations of Yunnan, China. It is therefore necessary to increase the surveillance of specific risk groups and raise awareness about the possible infectious diseases to help limit the HEV transmission here.

## Introduction

Hepatitis E virus (HEV) is a non-enveloped virus with a single-stranded RNA genome of approximately 7.5 kb and has been classified under the *Orthohepevirus* genus within the *Hepeviridae* family [1]. It is considered a major cause of acute viral hepatitis (AVH) [2]. Although HEV infection is usually a self-limiting disease [3], HEV infection in pregnant women can result in severe acute hepatitis or severe complications involving abortion, premature delivery, death of a live-born baby soon after birth, and can sometimes progress to fulminant hepatic failure (FHF) [4]. In addition, recent studies have highlighted that HEV infection is associated with renal injury, acute pancreatitis, hematological diseases, and several neurological disorders [5, 6]. Thus, HEV infection is a global public health concern.

HEV is transmitted efficiently through water, food, and the fecal-oral route [7]. Recently, HEV distribution in the world was shown to exhibit distinct epidemiological patterns, dependent on socioeconomic conditions, sanitation level, potable water, blood transfusion, and occurrence of zoonotic transmission [8]. However, zoonotic spread has recently become the predominant mode of transmission of hepatitis E in China [9]. Zoonotic HEV infections in humans occur via consumption of raw or undercooked meat, raw liver, and sausages; vocational contact; or livestock excreta that results in environmental contamination of agricultural products and seafood [8, 10].

Hepatitis E is more common in developing countries than in the developed countries. China was reported as one of the regions with high HEV infection prevalence and hepatitis E outbreaks occurred in Xinjiang during 1986 to 1988, with a total of 119,280 cases and 707 deaths [11]. Epidemiologic data from China indicate that the prevalence of anti-HEV IgG among different populations was 11% to 49%, including 11.1% in pregnant women [12], 23.5% in general population [13], 26.7% in veterinarians [14], 29.2% in blood donors [11], 39.7% in HIV-infected patients [13], and 48.25% in swine farmers [15].

Previous studies have reported higher anti-HEV IgG prevalence in provinces located in southern China [16], implying that geographical region is an important factor for HEV infection. Yunnan, located in southwestern China, has the highest number of ethnic groups. Twenty-five recognized ethnic groups are found here, and about 38% of the region's population belongs to members of the minorities [17]. However, the prevalence of HEV infection among ethnic populations remains unclear. The present study aimed to investigate the seropositive rate and risk factor for HEV infection and its impact on blood biochemistry in 1912 individuals belonging to different ethnic populations in the rural Yunnan province of China. The resulting data will contribute to a better understanding of the sero-epidemiology of HEV infection in this region and help to develop effective measures to better control HEV infections in the future.

## Materials and methods

### Study population

**Fig 1:**
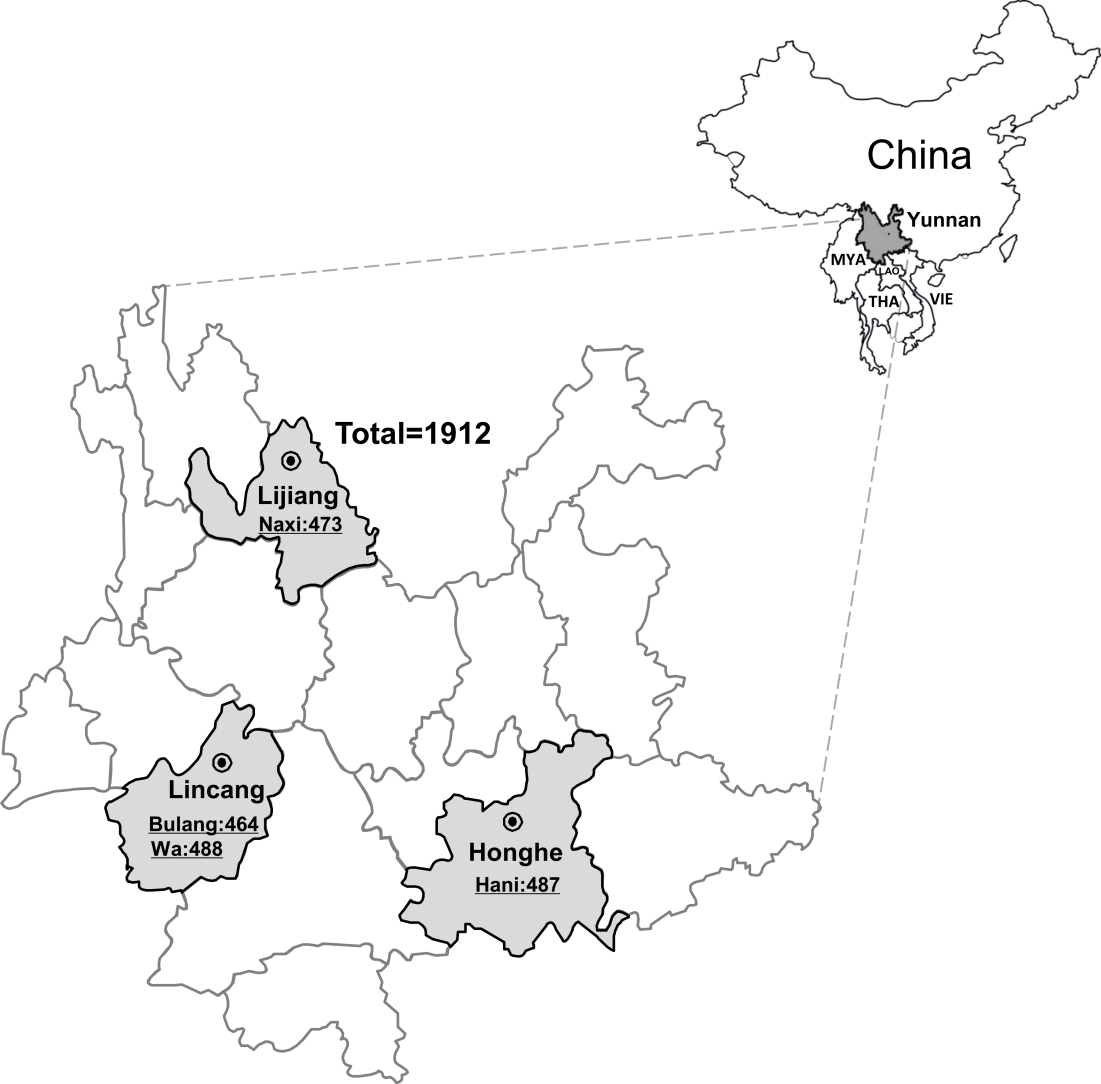
Maps of the study region and geographical distribution of subjects from all three prefectures of the Yunnan province of southwestern China. The Yunnan province of southwestern China is marked in dark gray, the Lijiang prefecture, Lincang prefecture, and Honghe prefecture are highlighted in light gray, respectively. MYA: Myanmar; THA: Thailand; LAO: Laos; VIE: Vietnam.

Serum samples were collected from 1912 individuals from four different ethnic minority groups (Wa, Bulang, Hani, and Naxi) residing in three different prefectures, Lijiang, Lincang, and Honghe, in the year 2015, in Yunnan, China. Of these, 473 cases were from Naxi ethnic, 464 from Bulang ethnic, 488 from Wa ethnic, and 487 from Hani ethnic (Fig 1). A random sample of residents aged 15 years or older was selected from the rural register in Yunnan. The sample was stratified by age and ethnicity, according to the identity card information. Demographic information and data on risk factors for HEV transmission were collected via a questionnaire (S1 and S2 Tables). Moreover, the standard clinical biochemical profiling, including test for liver function, renal function, blood glucose, and blood fat was performed at the time of sampling. The remaining serum samples were aliquoted and stored at -80°C, until further testing.

### Ethical statement

Informed consent was obtained from all the subjects before their participation in the study. The study was approved by the Kunming Medical University Ethics Committee. In those under 18 years old, the need for informed consent was explained by the investigator to each participant whose mother or father then signed it in on his/her behalf.

### HEV testing

Serum samples from participants were tested for antibodies against HEV (anti-HEV IgG and anti-HEV IgM) using an enzyme-linked immunosorbent assay kit (Wantai, Beijing, China), according to the manufacturer’s instructions. Serum samples positive for anti-HEV IgM were tested for HEV RNA by nested reverse transcription-PCR (nested RT-PCR), according to a previously published protocol [18].

### Statistical analysis

Statistical analyses were performed using the SPSS 21.0 statistical analysis software package. Data are presented as the mean ± SD. Univariable and multivariable logistic regression modeling was performed to analyze the risk factors for HEV IgG seropositivity. The nonparametric Mann Whitney t test was used to compare blood biochemistry data. p values below 0.05 were considered to indicate statistical significance.

## Results

### Demographic characteristics

We screened 1912 participants from three different prefectures (Lijiang, Lincang, and Honghe) of the Yunnan province for the presence of anti-HEV IgG and anti-HEV IgM antibodies (Fig 1). The ratio of males to females was 800:1112. The age of these participants ranged from 15 to 98 years, with a mean age of 48.79 ± 14.64 years. Of the 1912 participants, 25.5% (488) were Wa ethnic, 24.7% (473) were Naxi ethnic, 25.5% (487) were Hani ethnic, and 24.3% (464) were Bulang ethnic. Moreover, the different education levels of the participants were recorded as follows: Illiterate (746/1912, 39.0%); Primary (713/1912, 37.3%); Secondary (350/1912, 18.3%); and College and above (103/1912, 5.4%). For socio-economic level, 72.9% (1393/1912) belonged to the low-income group and 27.1% (519/1912) to the medium-income group. The demographic and epidemiologic data of study participants are shown in S3 Table.

Considering that age may be a very strong confounding factor for HEV seropositivity, the age distributions among the four different minorities are summarized in S1 Fig. The mean age ± SD of the Bulang ethnic participations was 48.8±14.3 years, 49.2±14.1 years in the Naxi ethnic population, 48.7±15.1 years in the Bulang ethnic population, and 48.4±15.0 years in the Wa ethnic population, and t test showed that there was no significant difference between the four groups in the age distribution. In addition, the number of participants in the four age groups (≤30, 31–45, 46–60, and >60) was coincident approximately in the four different minorities (S1 Fig). Taken together, our results indicated that no bias was introduced in the age factor.

### Prevalence of anti-HEV antibodies

**Fig 2:**
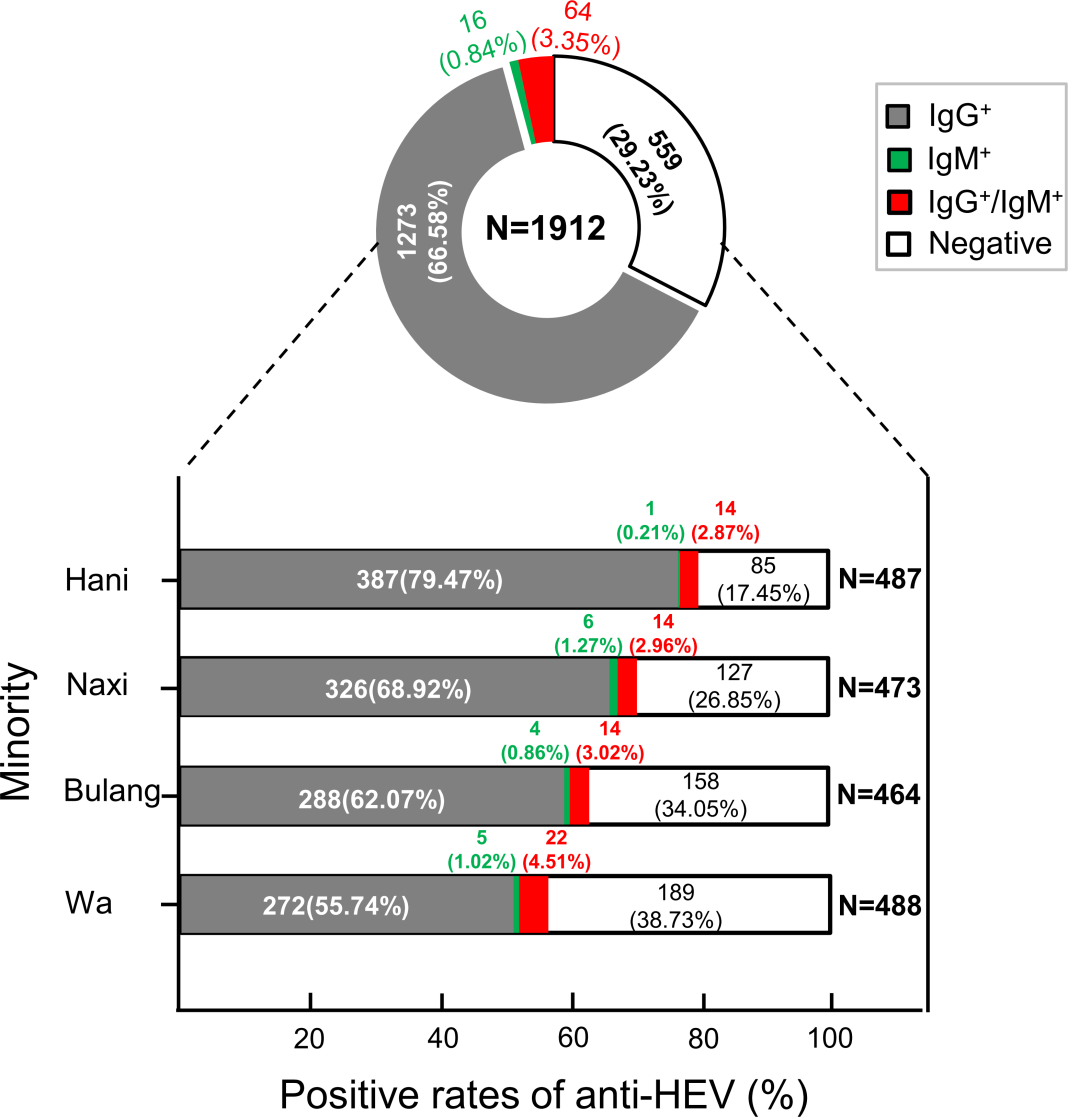
Comparison of anti-HEV IgM/IgG seropositity distributions among four different ethnic groups in Yunnan, China. The different seropositivities are shown in different colors, the anti-HEV IgG+ is marked in gray, anti-HEV IgM+ is highlighted in dark blue, and anti-HEV IgG and IgM co-positive is marked in red.

Of the 1912 plasma samples tested, 1273 (66.58%) were positive for anti-HEV IgG, 16 (0.84%) were positive for anti-HEV IgM, and 64 (3.35%) were positive for both anti-HEV IgG and IgM (Fig 2). However, none of the IgM-positive samples were positive for HEV RNA. The prevalence rates for IgG, IgM, and both were 79.47% (387/487), 0.21% (1/487), and 2.87% (14/487) in the Hani ethnic population; 68.92% (326/473), 1.27% (6/473), and 2.96% (14/487) in the Naxi ethnic population; 62.07% (288/464), 0.86% (4/464), and 3.02% (14/464) in the Bulang ethnic population; and 55.74% (272/488), 1.02% (5/488), and 4.51% (22/488) in the Wa ethnic population, respectively (Fig 2). The prevalence of IgG in the Hani, Naxi, and Bulang ethnic populations was significantly higher than that in the Wa ethnic population (p < 0.001).

### Risk factors for HEV infection in ethnic minority populations

To investigate the risk factors for HEV infection in the study cohort, only the samples with the most common HEV antibody serotype, i.e., IgG-seropositive samples, were analyzed; samples positive for IgM and both IgG and IgM were not included because of the limited number of individuals. Univariable analysis revealed that HEV IgG prevalence was associated with gender, age, ethnic origin, education level, smoking habit, body fat ratio, and visceral fat index (Table 1). In multivariate analysis, gender, age, and ethnic origin remained independent predictors for anti-HEV IgG seropositivity. HEV IgG prevalence in males was significantly higher than that in females (p = 0.001). The prevalence increased with increasing age and was the highest (77.9%; 328/421) in individuals above 60 years (p < 0.001). Moreover, there was a strong statistical association between ethnic origin and HEV IgG seroprevalence. Anti-HEV IgG reactivity in the Hani ethnic (82.3%; 401/487) population was higher than that in the Naxi (71.9%, 340/473), Bulang (65.1%; 302/464), and Wa (60.2%; 294/488) ethnic populations (p < 0.0001).

**Table 1:** Results from logist regression of anti-HEV IgG seropositity.

Variable	No.tested	HEV IgG seropositive		Univariate Analysis		Multivariate Analysis	
		n	%(95% CI)	OR (95% CI)	p-value	OR (95% CI)	p-value
**Gender**							
Male	800	588	73.5 (70.4–76.6)	1			
Female	1112	749	67.4 (64.6–70.1)	0.744 (0.609–0.909)	0.004	0.707 (0.572–0.875)	0.001
**Age**							
≤30	235	104	44.3 (37.9–50.6)	1			
31–45	561	364	64.9 (60.9–68.8)	2.327(1.706–3.174)	<0.001	2.123(1.541–2.925)	<0.000
46–60	695	541	77.8 (74.8–80.9)	4.425(3.234–6.055)	<0.001	4.013(2.903–5.548)	<0.001
>60	421	328	77.9 (73.9–81.9)	4.443(3.145–6.275)	<0.001	4.489(3.107–6.485)	<0.001
**Ethnic**							
Wa	488	294	60.2 (55.9–64.6)	1			
Bulang	464	302	65.1 (60.7–69.4)	1.230(0.945–1.601)	0.123	1.336(1.015–1.758)	0.039
Naxi	473	340	71.9 (67.8–75.9)	1.687(1.287–2.210)	<0.001	1.571(1.175–2.100)	0.002
Hani	487	401	82.3 (79.0–85.7)	3.077(2.290–4.133)	<0.001	2.969(2.188–4.030)	<0.001
**Education Level**							
Illiterate	746	553	74.1 (71.0–77.3)	1			
Primary	713	492	69.0 (65.6–72.4)	0.777(0.618–0.976)	0.03		
Secondary	350	233	66.6 (61.6–71.5)	0.695(0.527–0.916)	0.01		
College and above	103	59	57.3 (47.7–66.8)	0.468(0.306–0.715)	<0.001		
**Socio-economic level**							
Low	1393	986	70.8 (68.4–73.2)	1			
Medium	519	351	67.6 (63.6–71.7)	0.862 (0.694–1.072)	0.182		
**Drinking**							
No	1130	780	69.0 (66.3–71.7)	1			
Yes	782	557	71.2 (68.1–74.4)	1.111 (0.910–1.356)	0.302		
**Smoking**							
No	1279	864	67.6 (65.0–70.1)	1			
Yes	633	473	74.7 (71.3–78.1)	1.420 (1.146–1.759)	0.001		
**Labor intensity**							
Normal	688	467	67.9 (64.4–71.4)	1			
High	1224	870	71.1 (68.5–73.6)	1.163 (0.950–1.424)	0.143	1.399 (1.109–1.765)	0.005

**Education level,** IIIiterate: no education; Primary: 1–6 years of education; Secondary: 7–9 years; College and above: more than 9 years.

**Socio-economic level,** Low: less than 30,000 RBM of average annual income; Medium: 30,000–50,000 RBM.

**Drinking,** No: never or occasionally drink; Yes: often or every day.

**Smoking,** No: never or occasionally smoke; Yes: often or every day.

**Labor intensity,** Normal: no exhaustion; High: exhaustion.

### Blood biochemical characteristics of HEV-infected patients

Biochemical analysis of the blood samples showed that individuals positive for HEV IgG had significantly higher levels of total and direct bilirubin (TBIL and DBIL) and alanine amino transferase (ALT) but significantly lower levels of globulin (GLO) and low-density lipoprotein, than those negative for HEV IgG (Table 2).

**Table 2:** Blood biochemical characteristics of ethnic minority populations acoording to anti-HEV IgG status.

Biochemistry indexes		Anti-HEV IgG positive N = 1337	Anti-HEV IgG negative N = 575	p-value
**Liver function**	Total bilirubin (umol/L)	6.59 ± 4.14	6.34 ± 3.94	**0.0408**
	Direct bilirubin (umol/L)	3.07 ± 1.55	2.90 ± 1.50	**0.0025**
	Indirect bilirubin (umol/L)	3.52 ± 2.93	3.44 ± 2.79	0.5504
	Total protein (g/L)	73.67 ± 5.36	73.87 ± 5.24	0.4797
	Albumin (g/L)	41.73 ± 3.18	41.45 ± 3.15	0.0554
	Globulin (g/L)	31.93 ± 3.63	32.43 ± 3.47	**0.0062**
	A/G	1.32 ± 0.17	1.29 ± 0.16	**<0.0001**
	Alanine amino transferase (U/L)	15.47 ± 9.70	14.58 ± 9.28	**0.0366**
	Aspartate amino transferase (U/L)	27.60 ± 16.61	27.13 ± 14.66	0.9837
	AST/ALT	2.29 ± 1.71	2.44 ± 2.09	0.0282
	Alkaline phosphatase (U/L)	78.34 ± 26.40	78.74 ± 26.96	0.9626
	Glutamyl transpeptadase (U/L)	46.18 ± 91.40	43.81 ± 73.38	0.2702
**Renal function**	Urea (umol/L)	5.16 ± 1.44	5.12 ± 1.47	0.5257
	Creatinine (umol/L)	73.28 ± 14.38	73.06 ± 14.70	0.7119
	Uric acid (umol/L)	256.59 ± 66.02	257.85 ± 66.71	0.8172
**Blood glucose**	Glucose (mmol/L)	4.83 ± 1.03	4.80 ± 1.15	0.1494
**Blood fat**	Total cholesterol (mmol/L)	4.88 ± 1.04	4.92 ± 1.01	0.3903
	Triglyceride (mmol/L)	2.14 ± 1.51	2.06 ± 1.44	0.2492
	High density lipoprotein (mmol/L)	1.34 ± 0.31	1.34 ± 0.31	0.8038
	Low density lipoprotein (mmol/L)	2.77 ± 0.81	2.85 ± 0.83	**0.0436**

## Discussion

HEV is a small RNA virus that causes acute viral hepatitis and is transmitted enterically. Although HEV infection has been reported to be higher in southern regions of China, the HEV infection in the ethnic population of China is largely unknown. In the present study, we evaluated the seropositive rate, risk factors, and clinical impact of HEV infection in 1912 individuals belonging to ethnic minorities in rural Yunnan province of China. A high prevalence of anti-HEV IgG (66.58%) was found in the cohort studied, and this prevalence was comparable to that reported in Egyptian villages (67.7%) [19]. The prevalence revealed in this study is higher than that reported after similar investigations in the general population of the Netherlands (28.7%) [20], rural Taiwan (29.5%) [21], rural Mexico (36.6%) [22], and Cambodia (18.4%) [23], but lower than the seroprevalence of HEV infection among pregnant women in Egypt (84.3%) [24]. Moreover, the overall prevalence rate of anti-HEV IgM was 0.84% in our study, implying a low level of recent infection, which is in accordance with other previous reports stating that HEV IgM prevalence is about 0.5–5% among healthy blood donors [11]. HEV RNA was not seen in any of the anti-IgM–positive serum samples. These findings are consistent with the results from previous studies [12, 20, 25]. This may be attributed to the low viral load and/or the transient existence of viremia in the serum samples.

The present study demonstrated that prevalence of anti-HEV IgG increased with increasing age, and was significantly higher in men than in women; these findings are in agreement with several previous studies [8,20,21,26], suggesting that as a general rule, older age and male sex were independently associated with the risk of past HEV infection. This may be because these populations had cumulative exposure to HEV.

Previous study has demonstrated that the performance of anti-HEV IgG assays played a role in the difference of prevalence [26]. Among the anti-HEV EIA assay, Wantai assay has the most reliable estimate of the HEV seroprevalence [20] and greater sensitivity than others [8]. In addition, previously reported a lot of data of HEV seroprevalence were obtained from Wantai assay [11–15]. Given that the seroprevalence data in the HEV epidemiological studies should be compared, present studies using Wantai assays investigated HEV seroprevalence. Therefore, we presented the seroprevalence of HEV are more accurate and reliable.

Notably, we found a strong statistical association between ethnic origin and HEV IgG seroprevalence. Our data also indicate that this cohort had a significantly higher risk of being seropositive for HEV than that of other populations, including blood donors [11], HIV-infected patients [13], pregnant women [12], swine farmers [15], farmers [15], and veterinarians in China [14]. These findings imply that the ethnic minority population was more easily exposed to active HEV.

HEV mainly spreads via fecal-oral transmission through contaminated water and poor sanitation, zoonotic transmission, and foodborne transmission. Indeed, mixed farming of domestic animals is a common practice in Yunnan, China [27]. Almost all the farmers in the ethnic minority areas studied raise livestock and/or poultry such as pigs, cattle, goats, dogs, chickens, ducks, and geese in their homes. In fact, HEV-related viruses have been found in pigs, goats, chickens, and wild rats and several studies have demonstrated high prevalence of HEV infection in pigs, goats, and dogs in Yunnan [28, 29]. Another reason for high HEV IgG prevalence in the ethnic minority population may be the habit of eating undercooked or raw meat and drinking raw milk from cows and goats [30, 31]. It was recently reported that excretion of HEV into milk in cows imposes high risks of zoonosis [27]. HEV IgG prevalence was the highest in the Hani ethnic population (82.3%; 401/487) because of the habit of eating raw pork liver and fresh blood from pigs, goats, and dogs [32]. However, because the risk factors (eating raw meat, drinking raw milk from cows and goats, drinking water source, and so on) for HEV infection are not well defined, data for risk factor assessment could not be collected during the study; therefore we could not conclusively evaluate the route of transmission or the extent of zoonotic HEV infections and the role of consumption of uncooked or undercooked meats in HEV infections in the ethnic minority population. Further molecular epidemiological surveys are essential to monitor the epidemiology of HEV among the livestock and poultry and to collect more data, including that on occupation, raising farm animals, pet ownership, pork meat consumption, unpasteurized cow/goat milk consumption, and untreated water consumption, to determine the route of HEV transmission.

The present study indicates that high labor intensity is an important factor that increases HEV force of infection. In the rural areas of Yunnan, farmers' high strength manual labor is mainly concentrated in the breeding and planting of rice [33]. The participants with high strength manual labor usually are from poor families. Thus we speculate that high labor intensity is associated with an insanitary environment, such as insanitary water sources and food. An insanitary environment can increase the chance of contaminating water and food with HEV.

Furthermore, in the present study, we examined the biochemical liver functions, renal function, blood glucose, and blood fat, according to anti-HEV IgG status. Although there were significant differences in liver function indicators (TBIL, DBIL, GLO, and ALT) between anti-HEV IgG-positive group and IgG-negative group, these values were all within the normal range, indicating that the population was previously infected with HEV and showed no liver damage.

There were some limitations to this study. First, our study lacked a long-term follow-up epidemiological survey of HEV. Second, the information from questionnaires and interviews was limited; therefore, we could not comprehensively evaluate the risk factors. Third, the lack of HEV RNA detection made it impossible to analyze the HEV genotypes in the ethnic minority population of Yunnan, China.

## Conclusions

In conclusion, our data suggest that the seroprevalence of HEV infection among the ethnic minority population in Yunnan is extremely common. To the best of our knowledge, this is the first large-scale HEV epidemiological survey in Yunnan. It provides endemically representative data and describes the current situation of sporadic HEV infection in the ethnic minority groups in Yunnan. These findings highlight the urgency of increasing efforts aimed at monitoring and prevention of HEV and increase the awareness of this disease to better control HEV transmission here.

## Supporting information

S1 Table English version of the questionnaire in this study. (PDF)

S2 Table Chinese version of the questionnaire in this study. (PDF)

S3 Table Demographic characteristics of study participants. (DOCX)

S1 Fig The age distribution in the four different minorities. The mean age ± SD of the four different minorities was displayed at the top of the graph. The four age groups (≤30, 31–45, 46–60, and >60) of the different minorities are separated by red dotted lines. The number and percentage of participants of each minority is shown in different colors, Hani is marked in blue, Naxi is highlighted in green, Bulang is marked in black, and Wa is highlighted in red.
(TIF)
