# Supplementary material for: High seroprevalence of hepatitis E virus in the ethnic minority populations in Yunnan, China
Source: PLoS One. 2018 May 22;13(5):e0197577. doi: 10.1371/journal.pone.0197577 (PMC5963781; doi:10.1371/journal.pone.0197577)
Supplement: S1 Table — (PDF) [file pone.0197577.s001.pdf]

Questionnaire survey

Inquirer number:______________

Date: _____/______/______ (dd/mm/yy)

Investigation site: Yunnan province_________prefecture________village_______

一、Basic information

1 、Gender: ①man ②woman

2 、Date of birth: _______/________/______(dd/mm/yy)

3 、Age: ______years; height: ______cm; weight: ______kg;

4 、Marital status: ①unmarried ②married ③widowed ④divorced ⑤other

5 、Occupation: ①farmer ②teacher ③worker ④other

6 、Education Level: ①Illiterate ②Primary(1-6 years) ③Secondary(7-9 years)

④College and above (>9 years)

7 、Nationality: ① Hani ② Naxi ③ Wa ④ Bulang

8 、Labor intensity: ①Normal (No exhaustion) ②high (exhausted)

9 、Average annual income: __________RMB

二、Living and eating habits.

1 、Do you smoke tobacco?

① Yes(never or occasionally) ② No (often or every day)

2、 Do you have a drinking habit?

① Yes(never or occasionally) ② No (often or every day)

3、Do you keep a pet?

①Yes ②No

4、Do you raise livestock?

①Yes ②No

5、What is your source of drinking water?

① mineral water ② underground water

6、What food do you often eat? (multiselect)

①pork ②beef ③fish ④mutton ⑤Blood and/or guts ⑥vegetables ⑦other
