# Supplementary material for: High seroprevalence of hepatitis E virus in the ethnic minority populations in Yunnan, China
Source: PLoS One. 2018 May 22;13(5):e0197577. doi: 10.1371/journal.pone.0197577 (PMC5963781; doi:10.1371/journal.pone.0197577)
Supplement: S2 Table — (PDF) [file pone.0197577.s002.pdf]

云南少数民族问卷调查

询问者编号:______________

调查时间：________年 ______月 ______日

调查地点：云南省____________县___________乡（镇）_________村

一、基本情况

1 、性别：①男　　　②女

2 、出生日期：（以阳历计）______年______月________日

3 、年龄：_____ 岁； 身高：_______厘米； 体重：______ 公斤

4 、婚姻状况：①未婚　　　②已婚　　　③丧偶　　　④离异　　　⑤其他

5 、职业：①农民　　　②教师　　　③工人　　　④其他

6 、文化程度：①文盲　　②小学（1-6 年）　③中学（7-9 年）　④大专及以上 （>9 年）

7 、民族：①哈尼族　　②纳西族　　　③佤族　　　④布朗族

8 、体力劳动强度：①正常 （无疲惫感）　　②高 （有疲惫感）

9 、家庭年收入：___________元

二、生活和饮食习惯

1 、您是否吸烟?　　　①是（从不或偶尔）　　②否（经常或每天）

2、 您有饮酒习惯吗?　①是（从不或偶尔）　　②否（经常或每天）

3、您是否饲养宠物?　　①是　　　②否

4、您是否饲养家畜?　　①是　　　②否

5、您的饮水来源是?　　①矿泉水　　　②地下水

6、您经常吃以下哪些食物? （多选）

①猪肉　②牛肉　③鱼　④羊肉　⑤血、内脏　⑥蔬菜　⑦其他
