# Supplementary material for: High seroprevalence of hepatitis E virus in the ethnic minority populations in Yunnan, China
Source: PLoS One. 2018 May 22;13(5):e0197577. doi: 10.1371/journal.pone.0197577 (PMC5963781; doi:10.1371/journal.pone.0197577)
Supplement: S3 Table — (DOCX) [file pone.0197577.s003.docx]

| S3 Table. Demographic characteristics of study participants | |
| --- | --- |
| **Variable** | **Frequency (%)** |
| **Participants, n** | 1912 |
| **Gender** |  |
| Male | 800 (41.8) |
| Female | 1112 (58.2) |
| **Age** |  |
| ≤30 | 235 (12.3) |
| 31-45 | 561 (29.3) |
| 46-60 | 695 (36.4) |
| >60 | 421 (22.0) |
| **Ethnic** |  |
| Wa | 488 (25.5) |
| Naxi | 473 (24.7) |
| Hani | 487 (25.5) |
| Bulang | 464 (24.3) |
| **Location** |  |
| Lijiang | 473 (24.7) |
| Lincang | 952 (49.8) |
| Honghe | 487 (25.5) |
| **Education Level** |  |
| Illiterate | 746 (39.0) |
| Primary | 713 (37.3) |
| Secondary | 350 (18.3) |
| College and above | 103 (5.4) |
| **Socio-economic level** |  |
| Low | 1393 (72.9) |
| Medium | 519 (27.1) |

n, number.
