# Supplementary figures and images for: High seroprevalence of hepatitis E virus in the ethnic minority populations in Yunnan, China
Source: PLoS One. 2018 May 22;13(5):e0197577. doi: 10.1371/journal.pone.0197577 (PMC5963781; doi:10.1371/journal.pone.0197577)

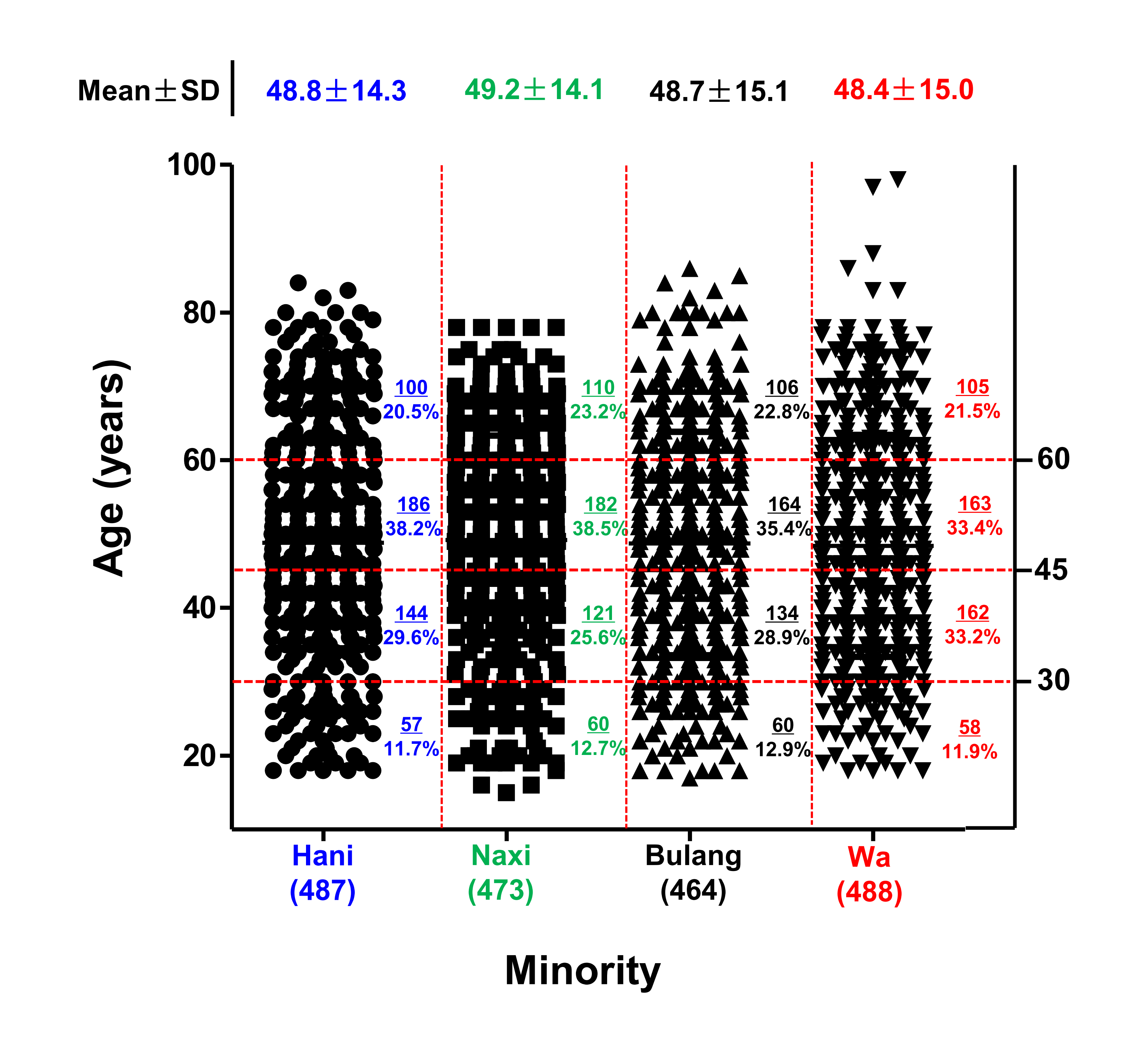

Supplement: S1 Fig — The mean age ± SD of the four different minorities was displayed at the top of the graph. The four age groups (≤30, 31–45, 46–60, and >60) of the different minorities are separated by red dotted lines. The number and percentage of participants of each minority is shown in different colors, Hani is marked in blue, Naxi is highlighted in green, Bulang is marked in black, and Wa is highlighted in red. (TIF) [file pone.0197577.s004.tif]
